# Polylactic Acid Piezo-Biopolymers: Chemistry, Structural Evolution, Fabrication Methods, and Tissue Engineering Applications

**DOI:** 10.3390/jfb12040071

**Published:** 2021-12-08

**Authors:** Amirhossein Farahani, Abbas Zarei-Hanzaki, Hamid Reza Abedi, Lobat Tayebi, Ebrahim Mostafavi

**Affiliations:** 1 Hot Deformation & Thermomechanical Processing Laboratory of High Performance Engineering Materials, School of Metallurgy and Materials Engineering, College of Engineering, University of Tehran, Tehran 11155-4563, Iran; 2 School of Metallurgy & Materials Engineering, Iran University of Science and Technology (IUST), Tehran 16846-13114, Iran; 3 School of Dentistry, Marquette University, Milwaukee, WI 53233, USA; lobat.tayebi@marquette.edu; 4 Stanford Cardiovascular Institute, Stanford University School of Medicine, Stanford, CA 94305, USA; 5 Department of Medicine, Stanford University School of Medicine, Stanford, CA 94305, USA

**Keywords:** polylactic acid (PLA), piezoelectric, crystalline structural evolution, texture, tissue engineering, thermomechanical process

## Abstract

Polylactide acid (PLA), as an FDA-approved biomaterial, has been widely applied due to its unique merits, such as its biocompatibility, biodegradability, and piezoelectricity. Numerous utilizations, including sensors, actuators, and bio-application—its most exciting application to promote cell migration, differentiation, growth, and protein–surface interaction—originate from the piezoelectricity effect. Since PLA exhibits piezoelectricity in both crystalline structure and an amorphous state, it is crucial to study it closely to understand the source of such a phenomenon. In this respect, in the current study, we first reviewed the methods promoting piezoelectricity. The present work is a comprehensive review that was conducted to promote the low piezoelectric constant of PLA in numerous procedures. In this respect, its chemistry and structural origins have been explored in detail. Combining any other variables to induce a specific application or to improve any PLA barriers, namely, its hydrophobicity, poor electrical conductivity, or the tuning of its mechanical properties, especially in the application of cardiovascular tissue engineering, is also discussed wherever relevant.

## 1. Introduction

Active materials, intelligent or smart materials, and adaptive materials all have similar definitions in that the materials are capable of utilizing one or more properties together in response to an externally applied stimulator. The most popular are shape memory alloys, electrostrictive and magnetostrictive materials, optical fibers, electro-rheological materials, and piezoelectrics.

The word piezoelectricity (PE) is derived from the Greek word meaning pressure electricity. Because of a wide bandwidth, a fast electrochemical response, low power requirements, and high generative forces, PE materials are among the most widely used smart materials. The subset of PE is pyroelectricity; ferroelectricity is a property of certain dielectrics that exhibit polarization from themselves, which is directionally reversible by applying an appropriate electric field. The origin of ferroelectricity is the division of the positive and negative charges into two poles and the non-neutralization of the total charge.

In recent years, however, researchers have explored the possibility of ferroelectricity in amorphous materials such as polymers.

PE materials have been popularly studied—but not wholly inspected—for energy harvesting, power generation, actuators, speakers, strain sensors [1], touch panels, screen instruments, and so on. PE is defined as the generation of electricity as a result of applying mechanical stress [2]. Electric fields are created without an external power source in piezo materials; nevertheless, control of the stimulation barriers is relevant in this field of research. Various therapy approaches, such as doctoring medicines, microfracture methods, allografting, and autografting, have been utilized to recover damaged tissue such as cartilage. Although these treatments aid in regenerating damaged cartilage tissue, restrictions also accompany them in most cases. Doctoring medicines require lasting rehabilitation, and the reconstruction of damaged tissue to its normal function is only partially possible [3]. The microfracture method has the potential to extract the subchondral bone marrow ancestor cells and repair the injuries, yet it is only suitable for minor injuries [4]. While maintaining all the mentioned positive results, TE has been used and demonstrated as an alternative approach to address these treatments’ limitations [5,6,7,8,9].

One of the novel solutions of TE is the use of scaffolding to overcome the imperfection of living tissues. A scaffold is a medium with which to culture cells to provide the growth factors [10]. The tissue repair and regeneration processes necessitate multiple steps that work together to ensure operative recoveries, such as between electricity propagation and mechanical pulses. Because of the physiological movements inside the body, scaffolds fabricated from PE material actuates are attractive choices to convert mechanical movements into electrical stimulation and vice versa. Countless hybrid and singular structures have been identified as efficient scaffolds for naive cell growth. Among them, materials with piezoelectric properties have significant potential for TE and regeneration.

The implementation of PE materials into TE has provided a wide range of applications, namely, the blood–brain barrier, drug delivery, cancer treatments, cartilage [11], TE, dental applications [12], fixation implants, and bone screws. Other stabilization tools, such as pins, washers, and darts [13], as well as nerve applications [14], skeletal muscle [15], and cardiac tissue engineering (TE) [16] are mentioned in the literature.

Researchers have been trying to find a sustainable energy source using triboelectricity, PE, and electromagnetics. PE generators are studied more often because of their excellent electromechanical properties. There are two types of PE materials—one is based on ceramics, and the other is polymer-based. Ceramic materials have a high PE constant, but they are brittle and lack ductility, while polymer PE materials are flexible with good durability but a low PE constant [17]. In this regard, the most known piezo polymer is PVDF and its copolymer, which has high chemical resistance and high PE properties and is biocompatible, lightweight, and flexible.

In biomedical applications, some essential parameters should be available to support the use of PE materials, for instance, mechanical interactions between cells and the material surface caused by cell adhesion, motility, cytoskeletal organization, cell migration, differentiation, and morphogenesis of tissue [18]. Several studies have shown that some cell lines require external stimuli to improve tissue repair, which is why the use of electro/mechanically active materials is increasingly gaining attention. Micro- and nanotechnologies have been used to help produce TE scaffolds and to provide more suitable environments for tissues to potentially regenerate by controlling the morphology and chemistry at the micro- and nanoscales. Other studies have shown that some cell lines, such as osteoblasts and chondrocytes, depend on external stimuli (mechanical or electrical) for enhanced tissue repair. Indeed, TE has also been used with active materials, such as conducting polymers and PE materials, to fabricate optimal scaffolds for specific applications.

## 2. Piezoelectricity in Polymers

### 2.1. The Origin of Piezoelectricity and Its Categorization

In 1880, the Curie brothers ascertained that when pressure is applied to quartz crystals and Rochelle salt, an electrical charge generates on the surfaces of these materials [19,20]. This discovery was a milestone in material science and engineering. Two different but related responses are possible with PE materials—direct and reverse piezoelectricity (PE). With applied mechanical stress, the response is direct. PE occurs when a dielectric material produces an electrical charge across its boundaries. Indirect or inverse PE occurs when the material shrinks or expands due to the employed electrical charge [2]. This property can be understood with the help of what is defined as a crystalline structure in material science and engineering—PE results from the non-inverse symmetric structure arrangement of the ions in a dielectric material [21].

**Figure 1:**
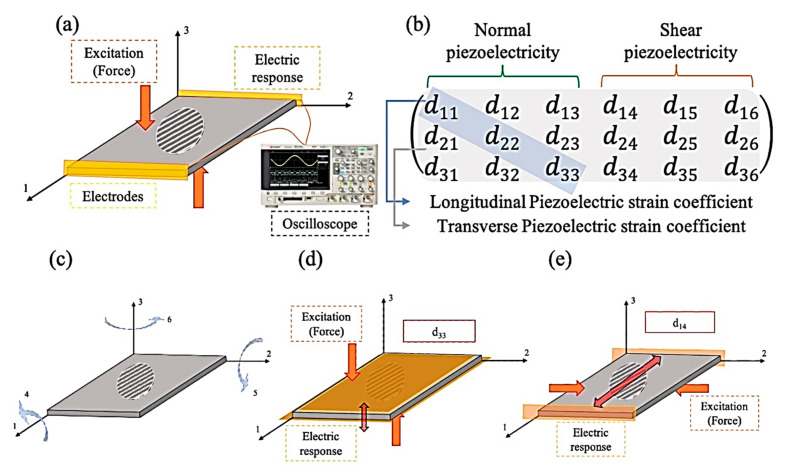
(**a**,**c**) Relationship between applied normal (σ) and shear (𝜏) stress and corresponding induced electric field (*E*), (**b**) piezoelectricity matrix, (**d**) d_33_ as an example of normal-longitudinal PE response, (**e**) d_14_ as an example of normal-longitudinal PE response.

There are two different types of PE—normal and shear. Normal PE is attributed to the materials that respond to the applied force directly. When the structure deforms in the X, Y, or Z directions, the electrical charge accumulates on one of the mentioned directions of the specimen. When an excitation charge is applied to the sample, volume accretion emerges in the same way. The shear PE definition is the material’s structural twist in response to the electrical stimulation [22]. A complete comprehensive schematic that illustrates PE is presented in Figure 1.

### 2.2. Ferro/Piezoelectricity in Semi-Crystalline Polymers

PE’s source in semicrystalline polymers becomes crystal clear when the focus is concentrated on the matter of polar domains in crystalline or amorphous regions. In such polymers, crystalline regions are dispersed in the amorphous matrix; this amorphous region has a glass transition temperature affecting the polymer’s mechanical properties; as a matter of fact, as the material’s T_g_ decreases, the elastic chains sliding over each other would be easier, and on the other hand, in the crystalline regions, the chain sliding is more complicated due to the newly ordered phases [23]. The degree of crystallinity in this type of material depends on its thermal history and method of preparation.

Several polymorphic phases come into account, which can be developed as a function of the crystallization conditions, focusing on semicrystalline polymers. It is noteworthy that not all of these phases are polar or thermodynamically stable, which is crucial when looking for stabilized piezoelectric material. Inducing a crystalline phase transformation, mechanical orientation, thermal annealing, and high voltage treatment are some methods that have a practical corollary [24].

Levo, a derivative of Chiral PLA Biopolymer, poly-L-lactide (PLLA) with 37% crystallinity, is reported for its high PE d_14_ = −10 pC/N constant [25]. Another study reports the d_33_ value of PLLA as 7–12 pC/N (smaller than PZT) and dielectric constant 2.5, making large PE output constant roughly similar to that of PVDF (both d_33_ and dielectric constants are high in this case). This advantage of the shear PE of PLA is applied in developing sensors as well [26].

Fundamentally, stretching the polymer aligns the amorphous strands in the film plane and aids in the consistent and uniform rotation of the crystallites by an electric field. The stretching will either be uniaxial or biaxial, so depending on this stretching, the electrical and mechanical properties will be either highly anisotropic or isotropic in the plane of the polymer sheet. Polymer poling can be accomplished using a direct contact method or a corona discharge. In this way, one of the popular methods is electrospinning (ES). During the ES process, fast evaporation of the solvent creates an amorphous structure of oriented fibers; it is much easier to increase the crystallinity by the hot drawing process. Surface area intensification, resulting from lower-diameter or more rough fibers, slightly decreases the glass transition and melting temperatures [27].

### 2.3. Ferroelectricity and Piezoelectricity

Although all piezoelectric materials are ferroelectric, not all ferroelectric materials are piezoelectric. Considering this issue, PVDF follows both piezoelectric and ferroelectric states, while PLA tends to be just a piezoelectric material lacking the ferroelectricity property. By applying a high electric field to semicrystalline polymers, for instance, PVDF polarization occurs nonlinearly, defined as a hysteresis loop. The proof of ferroelectricity is the reversal and spontaneous polarization. In this type of material, the quantity of the coercive field and the remanent polarization have to be adverted [28].

Inducing the PE to such ferroelectric polymers is governed by applying a high electric field during the fabrication method. This biased fabrication barrier is because the applied electric field should be near the permanent remanence, P_r_, to align the dipole domains, in PVDF case; C=F dipoles [29].

In this regard, the Curie-temperature T_c_ is close to the polymer’s melting temperature but lower. Below T_c_, the polymer is ferroelectric, and above T_c_, its non-centrosymmetric structure is disrupted. Although the ferroelectric phenomenon has been well acknowledged in ceramic crystals, the question of whether polymer crystallites could exhibit dipole switching was the source of many discussions for about a decade following the discovery of PE in PVDF [30].

## 3. Fabrication Methods

Fabrication of electroactive biopolymers contains various procedures, each encompassing advantages, and disadvantages. Both 3D and 2D porous scaffolds include essential functions meant to provide porous structures that are desirable for cell accommodation and activity. Numerous procedures have been employed to adjust porous scaffolds, namely, phase separation [31], gas-forming [32], freeze-drying [33], solvent casting/particulate leaching [34,35], and additive manufacturing [36].

These aforementioned methods possess problems related to the remaining toxic organic solvents, blocked pore structures, and unwanted skin layer formation. Even though the additive manufacturing technique can develop complex structures, it is not a cost-effective method, and only limited materials can be used in this technique [37].

On one hand, well-controlled pore structures are required; while on the other hand, the tuned mechanical property is essential for maintaining essential cell proliferation and matrix secretion to regenerate new tissue. Natural polymer-based scaffolds, such as processed collagen and gelatin scaffolds, provide numerous benefits, including bioactivity and biocompatibility, while their poor mechanical properties still remain as weakness.

Obtaining porous scaffolds is possible with some methods, namely, solvent casting/salt leaching, phase separation, gas foaming, gel casting, precipitation, and emulsion freeze-drying [38]. On the other hand, significant restrictions follow these methods, such as the probability of creating scaffolds with an inaccurate or restricted interconnectivity pore morphology that is disadvantageous for identical cell seeding and tissue extension [39], and their insufficiency to cause PE characteristics in the material.

The most attractive method for fabricating porous tissue mimetic artificial patches is the ES method, thoroughly discussed [40]. Fabricating excellent isotropic mechanical properties in PLLA samples with mold-based methods, such as high-pressure molding, injection molding, and vacuum molding, is among the conventional methods because of the prohibition of aligned chains and not arising the PE PLLA samples [41]. It is also possible to use 3D printing’s potential with molding methods [42]. In a study by Hayashi et al., they attempted to obtain orientated PLLA chains by optimizing the molding temperature and pressure to engender uniaxial-oriented molecular chains in a local area and promote PE [43].

Achieving nano/microfibrous structures mimicking the extracellular matrices (ECM) is an important consideration; several techniques have been introduced, namely, the self-assembly of peptide amphiphile, block copolymers, and dendrimers.

### 3.1. Electrospinning Parameters

Considering the technical issues, nanofibers with diameters between tens of nanometers to less than 1μm possess a high aspect ratio, which virtually leads to the definition of one-dimensional material. These peculiarities bring them unique properties, which are practical in various applications. Nanofibers are fabricated using numerous methods: wet-spinning [44], dry-spinning, or melt spinning [45,46]; template synthesis [47]; solution blow spinning [48]; and force spinning [49], to name a few. Still, the most prevalent highly effective procedure is the ES technique, i.e., the fabrication of nanofibers using electrostatic forces [50].

**Figure 2:**
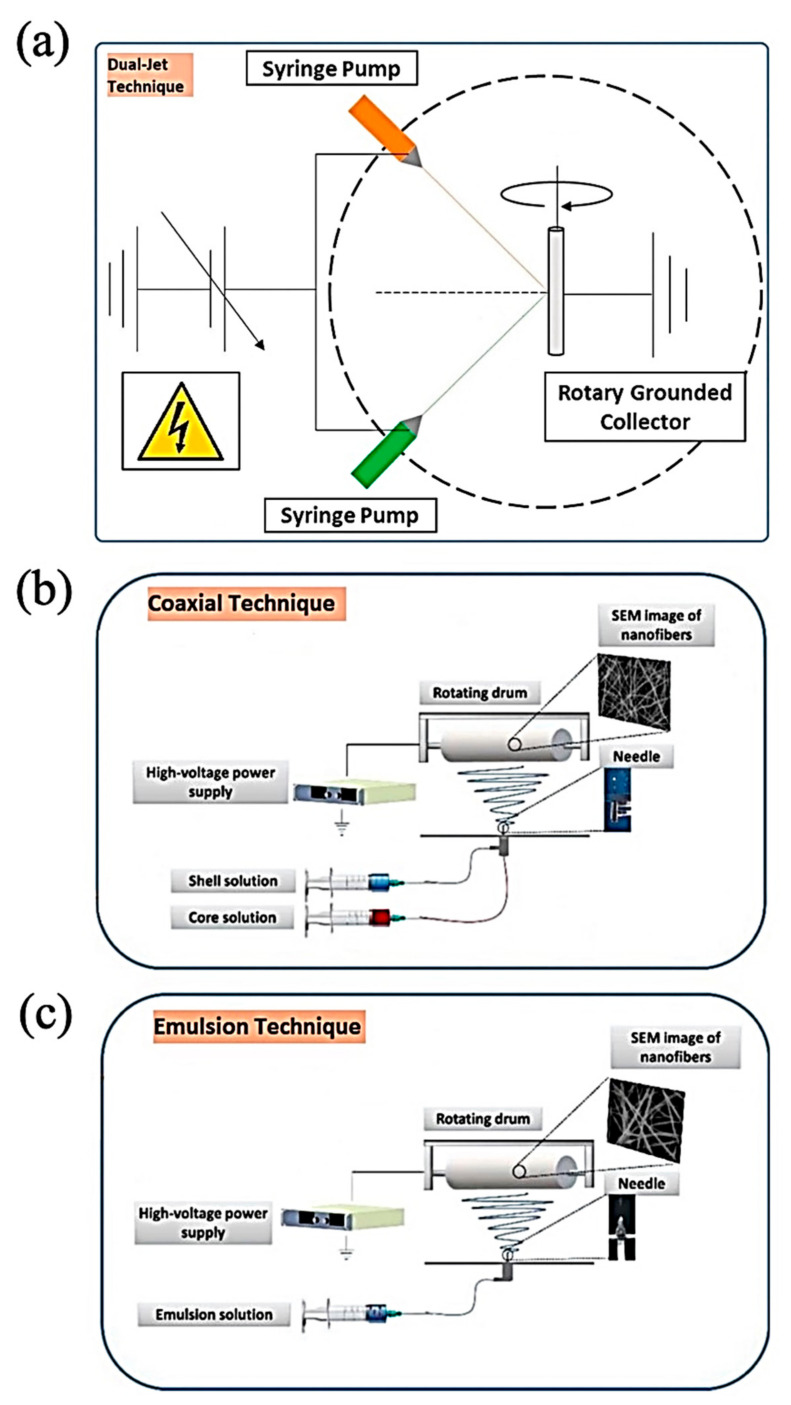
Schematic diagram of electrospinning: (**a**) Two-syringe technique, (**b**) Double channel nozzle technique, and (**c**) Single-nozzle technique; Adapted from [53], with permission from International Journal of Biological Macromolecules; Elsevier, 2021.

The process of ES (Figure 2) is essentially associated with its versatility; on the one hand, it is a mild and cost-effective manner, and on the other hand, the electrified polymeric jet develops instability and solidifies the fibers to design long, unbroken, and connected nanofibers with diameters differing from tens of nanometers to a few micrometers [51].

The physical and mechanical properties of the fibers can be varied as a function of different parameters such as solution concentration, solution feed rate, applied voltage, rate of needle movement, and the method of collection [52].

Research has proven the instrumental effect of electric fields during the ES process on the degree of disorientation of the molecular chain fibers and the molecular orientation, which increases by the increase in fiber orientation [54,55]. Optimizing the ES process for PLA has been extensively deliberated in previous studies, and affecting parameters on crystallinity, orientation, uniformity, thermal stability, diameter, and strength has been partially quantified. Utilizing a rotating collector drum in the ES setup is a common procedure to obtain oriented fibers, and it has been proven that, by changing the rotation speed, the fiber orientation can be tailored.

During the ES process, the mat thickness increases; this increase weakens the potential difference between the collector and the nozzle, and thus, the fibers’ alignment decreases. As the electrodes are covered with fibers, the electrostatic forces that aid the fiber orientation weaken, and it is easier to overcome the unpredictable whipping motion of the jet [56]. The process requires a very high voltage to turn a polymeric solution into thin fibers with desirable orientations. ES can be near-field or far-field depending on the type of application [57] and on the specific desired morphology.

The ES fibers can be deposited in various morphologies: aligned or randomly patterned and directly drawn to form ropes. Within this process, the fiber diameter and intermolecular dipoles causing an electrically induced strain are the points that stand to attention. Electro-spun fibers show a higher piezo constant than PLLA molded films; this is related to fibers’ ultimate uniaxial alignment [58]. One of the advantages of ES is the creation of uniaxially aligned dipoles because of the presence of electrostatic forces during fiber extrusion and the pulling toward the collector plate [59,60].

A study conducted by Luo et al. [61] introduces a method to select the best solvents for the ES process. Their work has proven that the best choice for ES is not necessarily related to the solvent with higher solubility, but also that balancing the electrostatic and fluidic forces should be taken into account to achieve smooth fibers. Therefore, solution conductivity, surface tension, and viscosity play essential roles.

Due to the material’s dielectric constant, the mass throughput, which is exposed to the electric field, takes effect. Possibly one of the most influential parameters in the ES solution is the charge distribution. Choosing the correct solvent is another effective parameter; Dimethylformamide (DMF) is a poor solvent containing significant dipole moments and has better conductivity than chloroform. Adding small amounts of DMF can improve the electrospinning ability and decreases the solubility. When increasing the amount of DMF, beading starts to appear, attributed to a decrease in polymer chain dispersion [62].

### 3.2. Electrospinning and Piezoelectricity

The most common piezoelectric constant of PLA is defined as d_14_. Through the experimental measurements of research from Occhiai et al. on 0-cut PLLA, carrying side along the *z*-axis, which was also their elongation axis, samples demonstrated that these cases had a d_14_ PE coefficient equal to 9.82 pC/N [2].

**Figure 3:**
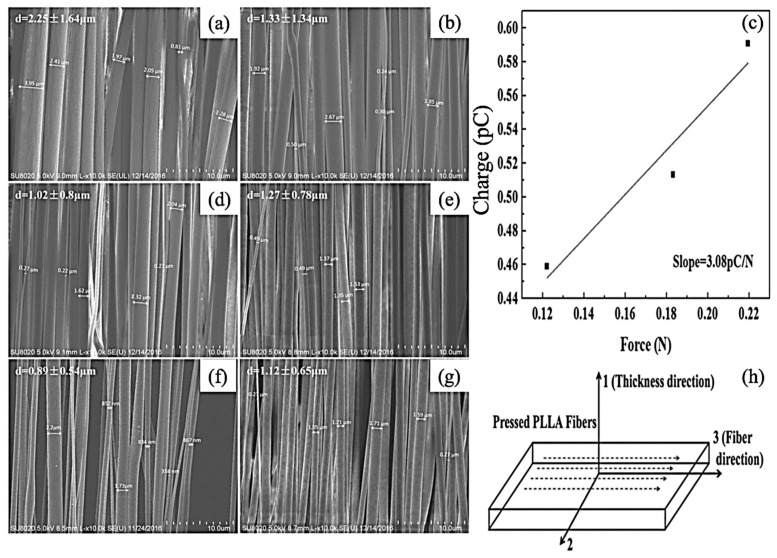
Scanning electron microscope (SEM) images of electrospun PLLA fibers fabricated at the distance of 10 cm from the needle to the rotational mandrel, a flow rate of 1 mL h^−1^: (**a**) 1000 rpm, 9 kV; (**b**) 1000 rpm, 12 kV; (**c**) 3000 rpm, 15 kV; (**d**) 2000 rpm, 9 kV; (**e**) 3000 rpm, 9 kV; (**f**) 3000 rpm, 12 kV. (**g**) Charge versus force plot for dynamic d33 test of the electrospun PLLA fiber bundles; (**h**) definition of coordinates for electrospun PLLA fiber bundles; Adapted from [63], with permission from Macromolecular Materials & Engineering; Wiley-VCH, 2015.

In an inceptive research, Zhu et al. fabricated porous electrospun 100 nm to 2 μm nanofibres. In another research [63], they found that optimizing the ES process led to the fabrication of higher than 800 nm PLLA fibers. Their method included a supercritical CO_2_ treatment. While they used melt drawing to fabricate the samples, scaffolds containing 40 μm nanofiber showed almost no piezo-response. However, aligned 100 nm to 40 μm nanofibers revealed piezoelectricity. The importance of this study was the reporting of d33 PE constant of a single polymer fiber. An increasing linear trend for the charge while increasing the force was observed, as it has shown in Figure 3.

**Figure 4:**
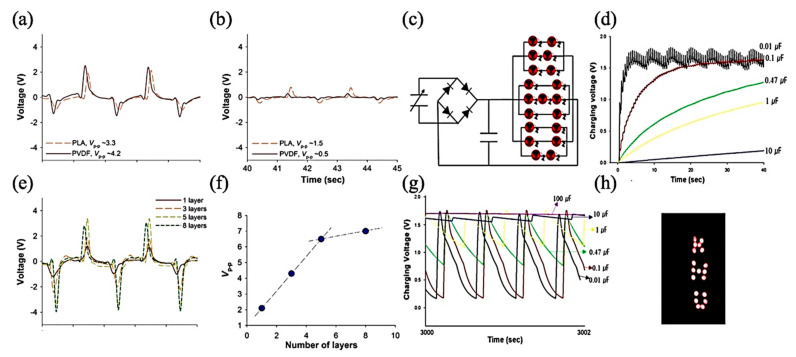
(**a**) Effect of constructive and (**b**) destructive stacking for PVDF and PLA nanofiber web-based sensors; (**e**) changes in piezoelectric output signal with increasing PLA nanoweb stacks and (**f**) its Vp–p comparison plot. (**c**) The equivalent circuit diagram to operate LED diodes; charging voltage vs. time when LEDs were (**d**) not connected and (**g**) connected for more than 3000 s for capacitors having different capacitances; and (**h**) photograph of LEDs operated using a 100 mF capacitor; Adapted from [64], with permission from Materials Letters, Elsevier, 2015.

Lee et al. studied the effect of constructive and deconstructive PVDF and PLA electrospun fibers [64]. Constructive PLA fabricated fibers show a slightly lower piezo-response than PVDF, while in deconstructive fabricated fibers, PLA shows a higher piezo-response than PVDF. To tackle the effect of the layers’ summation on the output voltage, they understood that by increasing the number of layers because of more dipole arrangement, the output voltage increases by a linear trend, and a similar result was reported for the current output. This research could use the fabricated PE to charge a capacitor to turn on an LED, as illustrated in Figure 4.

## 4. Poly Lactic Acid

### 4.1. Chemistry

**Figure 5:**
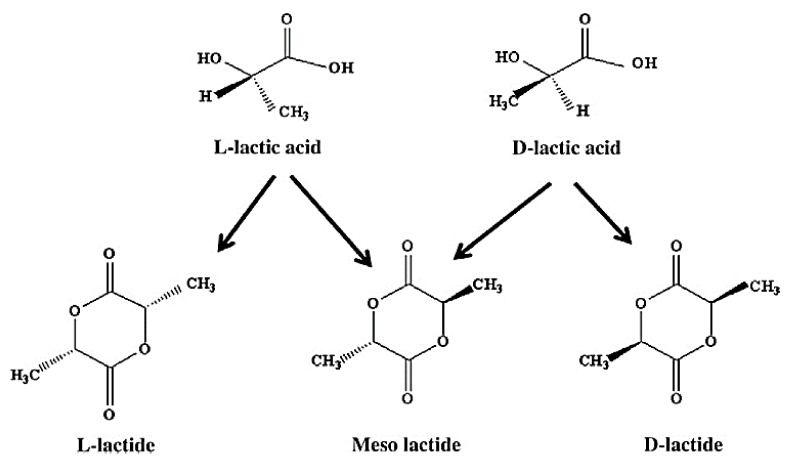
Polylactide acid stereo forms: L-lactide, D-lactide, and meso-lactide; Adapted from [66], with permission from Bioresource Technology; Elsevier, 2010.

PLA is produced from a natural organic acid, lactic acid, generated by the fermentation of sugars obtained from renewable supplies, such as sugarcane, and converts back to the latter when it hydrolytically degrades. Lactic acid (2-hydroxy propionic acid), CH3–CHOHCOOH, is a single chiral molecule existing as two enantiomers, L- and D-lactic acid, varying in the response of the polarized light. The optically inactive D, L, or meso form is an equimolar (racemic) mixture of D(−) and L(+) isomers [65], which is presented in Figure 5.

The discovery of PLA goes back to 1932, when a low-molecular-weight product was constructed by Carothers (DuPont), who performed this by heating lactic acid under vacuum conditions. Then, in 1954, DuPont produced and patented PLA with a higher molecular weight. Later, in 1968, Santis and Kovacs reported on the pseudo-orthorhombic crystal structure of PLLA. The PLLA crystal structure was reported as a left-handed helix confirmation for the α-form [67].

The PLA, which only contains L stereoisomers, is called PLLA; in contrast, when it contains only D stereoisomers, it is called PDLA. PDLLA is made of macromolecules containing both L and D stereoisomers (in different ratios). Both the PLLA and PDLA potentially have crystalline structures, whereas the percentage of PDLLA crystallinity depends on the L/D ratio. The L-isomer is a biological metabolite that forms the main portion of PLA and derives from renewable sources. Most lactic acid from biological sources exists in PLLA various phases (α, β, and γ), which will be discussed in detail in the following sections [68].

Materials with 3D configurations are categorized under the stereo complexation (SC) definition; this phenomenon occurs between isostatic and syndiotactic polymers, or L and D configurated polymers [69]. PLLA and PDLA have unique structures among this vast polymer class because of their high-performance biodegradable plastic properties [70]. The crystalline structure [71], crystallization, melting behavior [72,73], and mechanical properties [74] of PLA have been studied extensively.

Formatting a 1:1 mixture of high-PLLA molecular weight with a middle molecular weight of PDLA, the best annealing condition for SC was reported by Zhang et al. [75]. The best annealing temperature was considered in the melting temperature range (180 ºC) and SC (230 °C). The phase transformations may occur during the heat treatment of a specified polymer, where the crystalline phase melts-and-switches to another more thermodynamically stable state through the “melting and recrystallization process.” The development of SC crystals through the annealing of PLLA and PDLA single crystals was assumed by Fujita et al. to be prompted by both chain diffusion in solid-state and partial melting and recrystallization [76]. Xiong et al. discussed that the SC in the oriented PLLA/PDLA specimen takes root from the amorphous domains and molten crystals [77]. Molten α’ crystals crystallize to SC faster than the α phase because of the smaller aggregation and high SC nucleation density [78].

Poly (lactic acid) maintains a polymeric helix with an orthorhombic unit cell structure. The PLA features depend on various variables: the component isomers, processing temperature, annealing time, and molecular weight [66]. The stereochemistry and thermal history have a direct influence on PLA crystallinity and its properties. PLA containing higher than 90% PLLA content favors a crystalline structure, while the lower optically pure PLA tends to be amorphous. The melting temperature (T_m_) and the glass transition temperature (T_g_) of PLA are functions of numerous variables such as the L/D ratio, the PDLA macromolecules, crystallinity, chain alignment, confinement, etc. [79].

PLLA has attained glorious considerations because of its unique biocompatibility [80,81] and mechanical properties. However, its long degradation times, coupled with its high crystallinity, can cause erythrogenic reactions. Overcoming this drawback, PLA can be used as a material combination of L-lactic and D, L-lactic acid monomers. The latter is rapidly degraded without forming crystalline fragments during this process [82,83,84,85].

Generally, crystalline/amorphous polymer films consist of an aggregation of large molecular chains [86], with covalently bonded units [87,88,89]. PLLA has a complex higher-order structure of mixed crystalline and amorphous regions [90,91]. For example, in macromolecular materials, obtaining a 100% crystalline structure in macromolecular materials with conventional methods is impossible, and a proportion of an amorphous region is necessary because of the complexity of the structure [92].

### 4.2. Structural Evolution

#### 4.2.1. Crystalline–Amorphous Combination

Many semicrystalline polymers tend to have many polymorphic phases, and mechanical processes, thermal annealing, and high voltage treatment cause crystalline phase transformation by which various properties result. The degree of crystallinity/amorphous structure is defined as long/short-range ordering in the structure. It has been demonstrated that different properties such as the PE constant of PLLA are a function of the degree of its crystallinity and, of course, its molecular orientation [93,94]. Aligned electrical dipoles in the carbon–oxygen double bonds (C=O), which are branched out of the polymer backbone, are the main reason for PE [63,95].

Because of the formation of an amorphous sample based on the solvent’s inherent evaporation process, the pieces achieved by this would show a cold crystallization peak from themselves under DSC analysis. This fast solvent evaporation is an obstacle to crystalline phase formation. Choosing the correct temperature to form any further crystalline phases ought to bring attention to this T_cc_. The degree of stretching during hot drawing is related to the uniformity of fiber density and their alignment; another effective parameter is sweeping along the acrylic side [27]. Ribeiro et al. described these phenomena as frozen nucleation sites or non-equilibrium chain conformations and mentioned that spontaneous crystallization through the annealing process would be achieved [96].

Agrawal et al. [97] found a potential to increase crystallinity by the annealing process. A simultaneous combination of annealing and drawing allows a high proportion of polymer chains to crystallize under stress-controlled orientation [27]. The research explains that C=O polarization bonds might be canceled out along the amorphous PLA chain, resulting in no piezoelectric constant [98]. ES produces a metastable crystalline state and can orient the fibers [99], achieving a crystalline phase that needs post-processing.

The degree of crystallinity in PLA can vary between 0 and 50% by optimizing several factors, for instance, temperature, processing parameters, and solvents [96]. In research that has been conducted by Zong et al. [99], they found that PLLA fibers which were electrospun show much less crystallinity than the as-received PLLA but that cold crystallization occurred more efficiently due to molecular orientation. Thanh D. Nguyen et al. reported an annealing fabrication process to PLLA nanofibers to enhance the degree of crystallinity up to 80%. This work advanced the PE d_14_ constant of PLLA up to nearly 19 pC/N [98].

#### 4.2.2. Crystalline Phase Study; FTIR-XRD

One of the simplest PE PLA fabrication methods is based on the molding. Drawn PE PLLA films must conclude with chiral molecules forming a spiral in an arrangement along a single direction of the orthorhombic crystal. A molding procedure is not easy to align PLLA chain molecules along one axial direction [100,101]. As a result, the fabricated samples cannot exhibit PE from themselves or the most modest possible PE because of the polarity cancellation in the structure.

Considering the polymers, an individual polymer chain may be non-centrosymmetric. While stationing the amorphous network, these polymer chains will be in a highly graded isotropic state possessing a center of symmetry, which should be removed for the appearance of PE. This issue is solved chiefly by crystallization; among the 32 crystallographic point groups, 21 do not possess inversion symmetry, which is favorable for polymers to crystalline into one or two of them. In this way, multiple crystalline regions will nucleate and grow within the amorphous matrix and exhibit a random orientation distribution and show any level of preferential crystalline planes (texture) presence [102].

As a semicrystalline polymer, the crystallization process and crystal structure of PLA have been studied by various groups. PLA has the potential to form four kinds of crystal modifications, namely, α, α′, β, and γ [103,104,105,106,107], as a function of the preparation process. The most thermodynamically stable phase, the α form, has a 10_3_ helical chain conformation [108], which can crystallize from melt or solution and mainly differentiates from the α’ form. Based on the disorder to order phase transformation, the α’ form has been known to be the disorder form of α. The α’ form crystallizes at temperatures below 120 °C, while the α form achieves above that [105]. Table 1 epitomizes PLA crystalline phases’ information.

**Figure 6:**
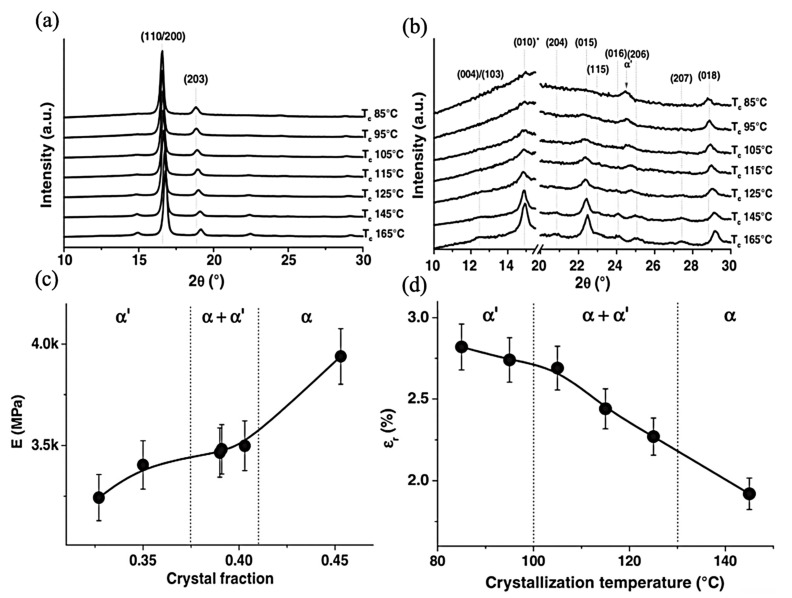
(**a**) XRD profiles of PLLA samples crystallized at different Tc; (**b**) Enlarged WAXD profile of PLLA samples crystallized at different Tc; (**c**) Young’s modulus of PLLA films as a function of the degree of crystallinity; (**d**) Elongation at break of PLLA films as a function of Tc; Adapted from [103], with permission from European Polymer Journal; Elsevier, 2011.

Cocca et al.’s fabrication technique was composed of compression-molded and quenched samples followed by an annealing process; they contended that by increasing the annealing temperature, the reflections of (110)/(200) and (203) planes shift to higher angles together with an increase in the intensity of the (010) and (015) planes. Furthermore, at the diffraction peaks at 2θ = 12.5, 20.8, 23, 24.1, and 25.1, they would appear at higher crystallization temperatures which are assignable to the reflections of the (004)/(103), (204), (115), (016), and (206) planes of α polymorphism, respectively. In comparison, they will not appear at temperatures lower than 95 °C. The formation of α’ could be attributed to this temperature. The α and α’ polymorphism is highly related to their molecular weight; in the reported paper, the construction of α is devoted to temperatures higher than 145 °C, and in the range of 105–125 °C, both α and α’ are able to be achieved at the 95 °C that is associated with α’. As shown in Figure 6, the research article also studies the correlation between the crystalline phases and the mechanical properties. The relation of Young’s modulus and crystal fraction increases but not in a linear trend for semicrystalline materials, resulting from the mobile amorphous fraction, which implies applied stress transformation across the amorphous–crystalline interface and can affect the response of semicrystalline structure [103].

Calculating the crystallite size (L) is available by Scherrer’s equation as it has been checked in the following parts; where β_1/2_ is the width of the peak at half intensity, λ is the wavelength of Cu–K_α_ radiation (1.5418 A°), which is primarily used in XRD characterization, and K is the broadening constant as 0.9 for imperfect polycrystal [106].

(1) $$L=\frac{K\times ƛ}{\beta_{\frac{1}{2}}\cos\theta }$$

The β form is obtained under a severe, high-temperature-drawing condition with left-handed frustrated 3_1_ helical conformations and encompassed of the trigonal unit cell with a = b = 1.052 nm, c = 0.88 nm [109,110], capable of accommodating the random up-down oriented neighboring chains [111,112], and finally, the γ can be achieved by an epitaxial crystallization route on the hexamethyl benzene substrate [113].

As mentioned previously, PLA is also polymorphic, with its corresponding α and α′ phases being the most common. The β-phase is found in drawn PLA samples in the same type of samples which are frequently used for PE analysis. This has made some researchers believe that the β-phase is necessary for the PE property [63]. While it is not correct, in this course, both the β-phase and PE are a consequence of drawing, and in this way, the presence of the β-phase is not a requirement for PE. PE is observed in samples drawn to a ratio of two [114], whereas significant proportions of the β-phase are not formed until draw ratios of at least four are achieved [115].

In the case of PLA identification, the orientation of PE crystallites must be performed by X-ray diffraction (XRD) [100]; however, because of the disturbance and low degree of crystallinity, it is hard to obtain a sharp pattern [116]. Therefore, controlling the structure of the PLA distributed in the amorphous region is important, while exhibiting macro-PE is expected. Smith et al. discuss the importance of the crystalline structure in polymers; they have proven the importance of a non-centrosymmetric structure on the appearance of the PE property.

**Figure 7:**
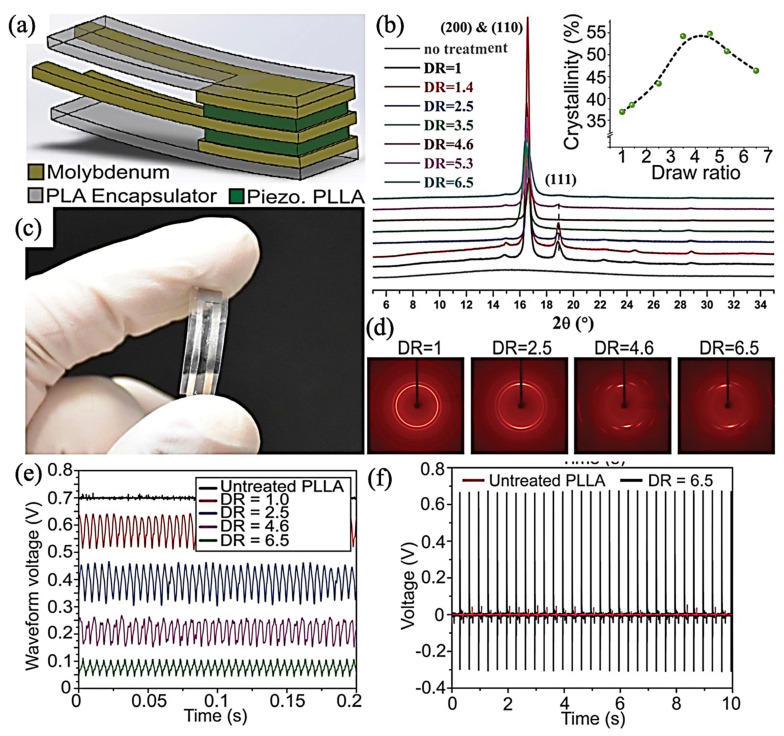
Biodegradable piezoelectric PLLA pressure sensor: (**a**) Simplified schematic representing the biodegradable piezoelectric PLLA sensor; (**c**) Optical image of a fabricated biodegradable piezoelectric PLLA sensor (5 mm × 5 mm and 200 μm thick). Characterization of crystallinity and polymer chain orientation for processed PLLA: (**b**) Results from one-dimensional (1D) XRD of stretched PLLA films with different DRs. (Inset) Crystallinity percentage of the processed PLLA for different DRs, quantified from the 1D XRD spectrum. (**d**) Two-dimensional XRD images show the polymer chain’s orientation of the stretched PLLA films with other DRs. Characterization of piezoelectric PLLA output from vibration and impact modes: (**e**) Voltage output from the treated PLLA with different DRs under a vibration at 200 Hz. (**f**) The voltage output from an untreated PLLA (red) and treated PLLA (black, DR = 6); Adapted from [117], with permission from Proceedings of the National Academy of Sciences of the United States of America, 2018.

Both of the α and α’ phases show (110)/(200) and (203) crystalline planes considering the XRD results while at 2θ = 16.8° and 19.1° and 2θ = 16.4° and 18.9° for α and α’, respectively. Low-intensity peaks at 2θ = 14.9° and 22.4° have been assigned for (010) and (211) α phase reflection, whereas such a weak reflection peak can just be found at 2θ = 24.6° for the (206) crystalline plane of α’ [112]. Cury et al. [117] had investigated the effect of the drawing ratio on PE properties of PLLA. The investigation on PLLA film showed that there are three crystalline phases which can be attributed to the (111), (200), and (110) crystalline planes. The intensity of (111) slumped by increasing the drawing ratio results from phase transformation from α, incorporating a left-handed 10_3_ helical conformation to β, with a 3_1_ helical conformation. As depicted in Figure 7, The optimum drawing ratio, which has the potential to maximize the PLLA film crystallinity, was five, and further elongations decrease the degree of crystallinity. This research used shear stress to monitor the generated electric potentials in drawn films. Because of crystalline phase transformation, films with drawing ratios between 2.5 and 4.5 showed the best PE response. Not considering the degree of crystallinity, uniaxial deformation has a promising potential to align the polar C=O polymer chain bonds and enhance PE.

Considering the crystallization from an amorphous state, FTIR characterization ought to have good potential to study the phase transformations; in this way, small bands at 921 cm^−1^ and 955 cm^−1^ wavenumbers are assigned to amorphous and α crystalline modes with 10_3_ helices, respectively, and the absence of 908 cm^−1^ peaks indicates the presence of the β form [113]. One of the essential PLA bindings is the C=O, which can be found around 1745 cm^−1^ for α and 1755 cm^−1^ for α’ [118].

PLA melt extrusion and subsequent warm stretching at 65, 90, and 120 °C at a constant drawing ratio of 5.4 m/min was utilized to study the α and α’ phases. Mi et al.’s research suggested that it is hard to identify two phases by 1D-XRD due to their similarity in diffracted peaks, but FTIR results are reliable in this course. The carbonyl stretching vibration bands showed a complex splitting pattern at 1776, 1759, and 1749 cm^−1^. In the α’ phase PLA film, the carbonyl stretching vibration bands had only a single peak at 1759 cm^−1^ [119]; it should be noted that the peak positions shift due to the processing method. Table 2 illustrates a vital conformation study on PLA C=O branched out bond.

**Figure 8:**
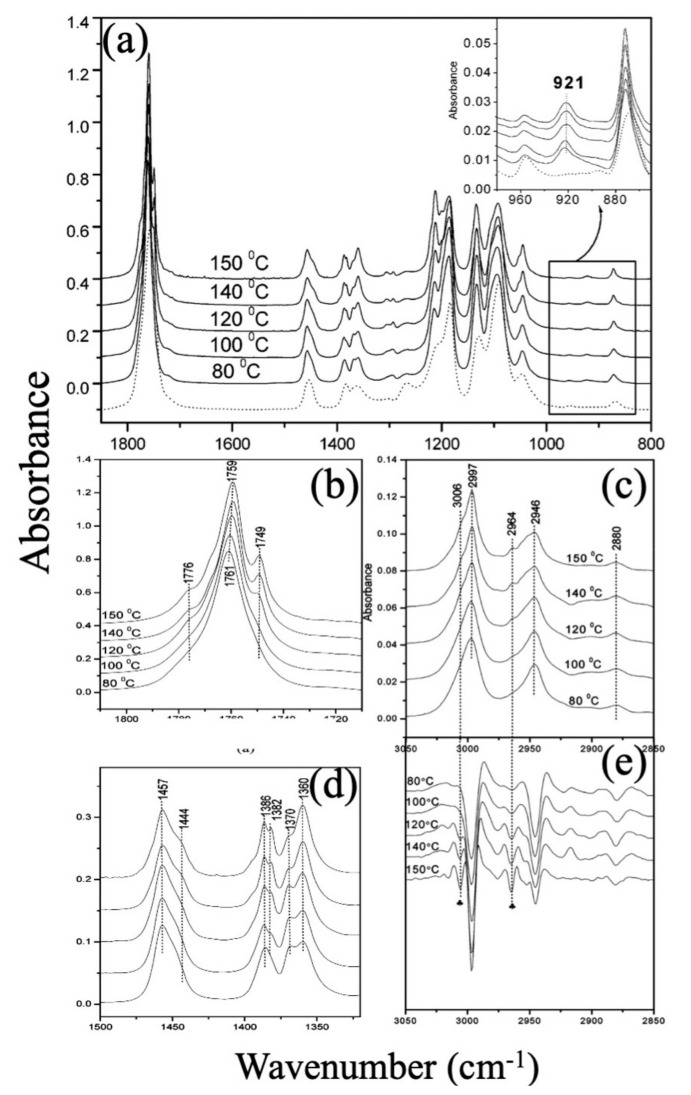
(**a**) IR spectra in the range 1850–800 cm^−1^ of various PLLA samples prepared by annealing at different temperatures from the amorphous state as indicated on the lines for 1 h; (**b**,**d**) Enlarged IR spectra of PLLA samples prepared by annealing at 80, 100, 120, 140, and 150 °C (**b**) 1810–1710 cm^−1^, (**d**) 1500–1320 cm^−1^; (**c**,**e**) IR spectra and the second derivatives in the 3050–2850 cm^−1^ region of PLLA samples prepared by annealing at 80, 100, 120, 140, and 150 °C; Adapted from [121], with permission from Macromolecules; American Chemical Society Publications, 2005.

Considering the IR spectroscopy technique, all of the PLLA samples show a band at 921 cm^−1^, which assigns to C–C backbone stretching with the CH3 rocking mode and is sensitive to the 10_3_ helix chain conformation of PLLA α crystals while the peak at 908 cm^−1^ is a denoted wavenumber for β polymorphism formation. The C=O stretching band appearing in the ranges of 1810–1710 cm^−1^ and 1500–1300 cm^−1^ is associated to CH3. Annealing above 100 °C can induce a new band appearance: (1) at 1749 cm^−1^, which is related to the C=O bond; (2) the band splitting of the CH3 asymmetric deformation mode around 1458 cm^−1^ and the CH3 symmetric deformation mode around 1386 cm^−1^; and (3) in the second-derivative spectra, two new high-frequency bands (3006 and 2964 cm^−1^) around the νas(CH3) and νs(CH3), respectively, become much clearer with the increasing crystallization temperature over 100 °C. As it is obvious in Figure 8, all in all, there are signs of α crystalline phase formation [121].

The physical crosslinking of macromolecular semi-crystalline PLA induces tensile drawing, and other thermo-mechanical processes will restrict PLA chains’ motion. Controlling polymer chain regulation affects the polymer microstructural evolution through stretching and annealing as a post-process [122]. The structural development through these types of designed procedures would make it possible to achieve various crystalline phases with different mechanical properties; for instance, γ isotropic crystallites toughened the sample by highly stretching the films concerning a high applied drawing ratio [123].

#### 4.2.3. Texturization Study

**Figure 9:**
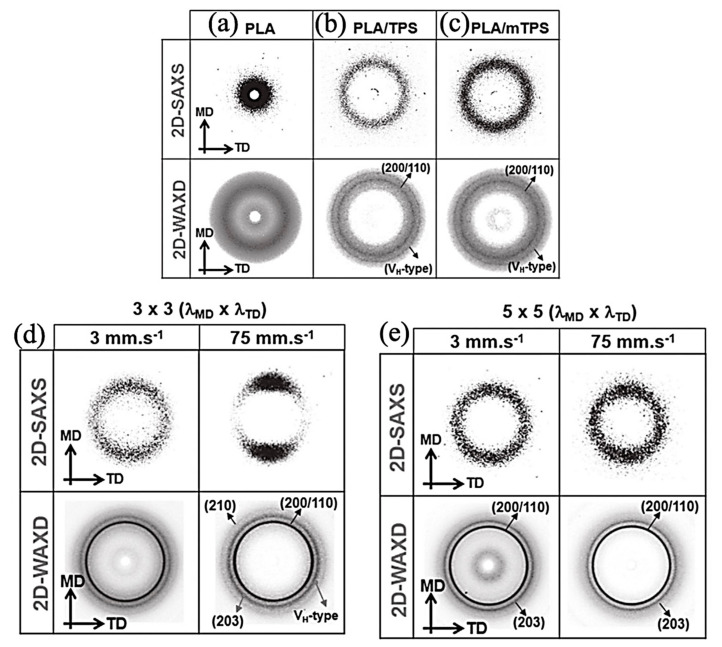
The 2D-SAXS and 2D-WAXD patterns of (**a**) PLA, (**b**) PLA/TPS, and (**c**) PLA/mTPS precursor sheets, respectively. (**d**,**e**) The 2D-SAXS and 2D-WAXD patterns of BO-PLA/TPS with varied stretching rates and draw ratios (3 × 3 and 5 × 5); Adapted from [124], with permission from Macromolecular Materials and Engineering; Wiley-VCH, 2019.

As Chirachanchai et al. [124] (Figure 9) observed, PLA shows a completely cloudy 2D XRD trend. While the addition of thermoplastic starch (TPS) has the potential to form PLA crystalline phase, a spot pattern, as a matter of TPS, has a nucleation role for PLA in addition to the rate and type of drawing ratio. Moreover,2D-WAXS is a pluripotent characterization technique to investigate the molecular orientation of the samples. It has been understood that the polymeric chains’ alignment is the function of thermo-mechanical processing. Both the strain and temperature are the functions that should be considered in this course. In research, it was indicated that the orientation inclination of the molecular chain occurred along drawing direction. It is demonstrated that at 65 °C and 90 °C, strong fiber texture developed progressively at drawing ratios above DR3. Herman’s orientation function investigates the degree of molecular alignment by the following equation [119]:

(2) $$f_{c}\;=\;\frac{3\left(\cos^{2}\alpha -1\right)}{2}$$

F_c_ is the orientation index and would be equal to 0 where there are randomly oriented chains and 1 where the chains are perfectly oriented, and cos^2^α is the average cosine value associated between the polymer chain axis and the drawing direction. The latter paper shows the value of f_c_ increased with increasing the drawing ratio and causing the chain alignment along the uniaxial draw direction due to strain-induced crystallization.

**Figure 10:**
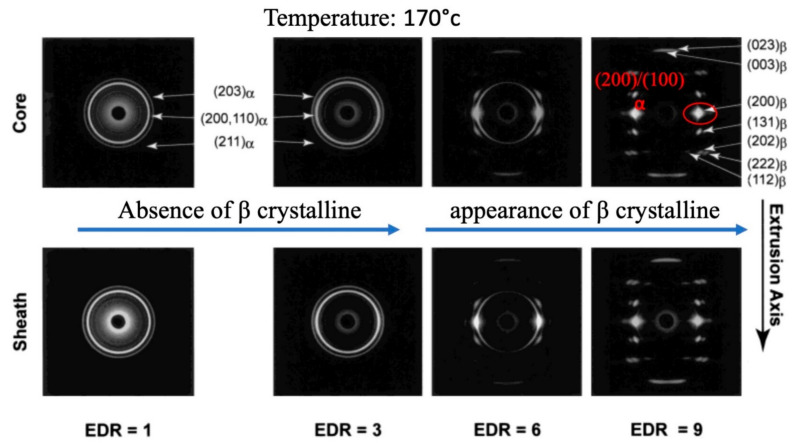
α to β crystalline phase transformation induced by different drawing ratios; Reproduced from [115], with permission from Macromolecules; ACS Publications, 2003.

In order to understand the PLLA crystalline structure phase transformation, mold casting at numerous temperatures and various drawing ratios was considered [115]. The best results to analyze were achieved when the temperature intervened between T_m_ and T_g_ at 170 °C. While the extrusion drawing ratio (EDR) was 1, 3, the α phase was achieved with any texturizing crystalline planes, while at higher drawing ratios of 3, 9, the β crystalline phase started to appear and texturization happened. The research denotes that the (200) of β phase crystalline plane coincides with appearing with (200)/(100) α at the lower drawing ratios, although at EDR = 11, the complete β crystalline phase is achieved with the absence of α. The results of this study are presented in Figure 10.

### 4.3. PLA Piezo-Polymer

Since the first discovery of the PE biopolymers in 1941 [106], PLA has emerged as the most attractive nominee to replace current PE materials. PLA offers more outstanding processability and a more comprehensive range of applications compared to PE-ceramics because of its flexibility and lightweight. It should be taken into account that the PLA PE strain-constant is remarkably low, and improving the PLA PE constant is the driving force of many researchers to make it a considerable alternative for conventional PEs [13,101]. PLA has unique piezo properties comparable with PVDF and other piezo-ceramics, affiliated with the chiral center of lactic acid [104,107].

In another point of view, PLA has not been classified under ferroelectric materials, and uniaxial alignment would act enough to implicate its inherent piezo-response. Accordingly, because of the inherent PE properties of the PLA, no other fabrication process other than stretching treatment, which is necessary to induce uniaxially oriented chains, is required. Inducing the PE is also possible by thermally stretching the polymer; by this process, the phase transition from α to β happens, so a change from randomly oriented molecular chains to aligned chains might be achieved [125].

A typical PLA thermoplastic biopolymer maintaining chirality and its Levo derivative, poly-l-lactide (PLLA) with 37% crystallinity, is reported for its high PE behavior (d_14_ = −10 pC/N) [25]. Another study reports the d_14_ value of PLLA as 7–12 pC/N and the d_33_ value as 0.21 fC/N for fabricated scaffolds [126]. (smaller than PZT) and dielectric constant 2.5, making large PE output constant roughly similar to that of PVDF (both d_33_ and dielectric constants are high in this case). This advantage of shear PE of PLA is applied in developing sensors as well [26]. Table 3 provides an appropriate comparison between various piezoelectric materials mentioned in the literature.

On the other hand, the existence of the lead, which leaches from the best performed PZT family PE, restricts its application potential [128]. The alternative group for PZTs is lead-free piezo-ceramics such as Barium Titanate Oxide (BTO) and potassium sodium Niobate (KNN)-based materials. The barrier of this group is the limitation of recycling and the non-environmentally friendly destruction products, along with the lack of flexibility [129].

In helical PLA molecule chains, shear strain in the direction of the helix axis slightly rotates the permanent bond dipoles and thus alters the polarization perpendicular to the plane of shear strain [114,130]. One of the drawbacks in this field is the PLA magnitude of PE, which is much lower than most other piezo-electrics. To overcome this drawback, scientists, on the one hand, started to fabricate hybrid biomaterials and, on the other hand, focused on phase transitions and stabilizing them. The convention magnitude between mechanical applied deformation and the electricity generated charge is a function of the PE constant [131].

Micro/nanoscale PE observations are possible by advanced characterization techniques [91,132]. For instance, Smith et al. reported the measurement of shear PE in highly oriented PLLA nanowires which were also highly crystalline; the analyzed result concluded an increase in the degree of crystallinity up to 70%, using PE Force Microscopy (PFM) [91]. In his study, nanowires were grown on a confined aluminum oxide template. They detected a sharp vertical response at the edge of the nanowires, denoting great shear PE. On the other hand, a significant deflection was detected in the lateral signal along the nanowires. The estimated d_14_ PE coefficient was about 8 pC/N.

An exciting potential in cell promotion, stimulation, proliferation, and differentiation is accessible using the biocompatible and biodegradable PLLA PE polymer. Drawn PE PLLA rods, in contrast to non-drawn PLLA rods with no PE response, form higher callus in the intramedullary cavity of the feline tibia of cats, fracture healing improves. The finding of this research shows the effect of PE PLLA drawn in fracture fixation devices [133].

### 4.4. Bio-Concern

Because of ester bonding in PLA chains when exposed to water or microbial milieu, hydraulic degradation happens, which comes from a bulk erosion mechanism by random scission of the backbone [17,134], Ishi et al. demonstrated in vivo degradation behavior of PLLA fibers. The research team implanted the fibers in Wistar rats; after 12 weeks [89], they confirmed the complete dissociation of the fiber structure and decreased the degree of crystallinity and molecular weight by in vivo biodegradation.

PLA is mainly used for applications, e.g., drug delivery and TE, which require biodegradable properties [135]. PLA is among the well-known and long-standing synthetic biopolymers. It has been demonstrated that the PLLA slowly degrades when reacting with the water environment of tissue [136]. In a study by Bos et al. [137], high-molecular-weight specimens implanted on the back of a rat and their in vivo long-term degradation was studied. A decrease in molecular weight was observed in a 3-month period. Continuous mass loss was observed up to 26 weeks with no chronic, acute inflammatory reactions, and after 104 weeks, macrophages appeared to clean the remaining PLLA content.

Curry et al. fabricated a PE force sensor which is completely biodegradable for the first time by a heat compression, mechanical stretching, annealing process at 90 °C for 8 h. PLLA fibers were cut at a 45° angle relative to the stretching direction to investigate the piezo properties. They incorporated molybdenum electrodes on PLLA film and encapsulated them with PLA to introduce a PLLA biodegradable implantable force/pressure sensor. The film was inserted into an incision below the mouse’s diaphragm, and with each mouse breath under anesthesia, a clear signal was detected and correlated to sinusoidal force nearly 0.1 N/cm^−2^. The best PE properties came from the drawing ratio in the range of 2.5–4.5, and the PE constant was approximately 11 pC/N. They observed the complete degradation of PLLA film in the phosphate-buffered saline (PBS) at 74 °C after 56 days [85].

It has been clear that through the degradation process, crystalline phases last more while amorphous regions disgrace faster. Here, it is worth noting that the PLLA can last for 5.7 years, maintaining its good biocompatibility up to its complete decomposing [138].

## 5. PLA Composites and Application

By expanding the science fringes, PE properties have shown their critical role in various engineering fields. It is expected for their market value to reach from 20.35 billion in 2015 to USD 27.24 billion by 2020, growing at a high compound annual growth rate of 6.01% [139].

The electronics industry is one of the significant industries looking to utilize renewable materials for replacing rare-metal-based and non-recyclable products. Electronic devices consist of organic or inorganic materials interfacing with human/animal bodies. Bio-piezo-polymers, which have no hazardous impact on the body, are the future of TE. They can function as sensors [140,141,142], actuators, in-vivo info processing systems, and biological collector signals [143,144,145,146,147,148]. PVDF is a chemically stable PE polymer with high piezo-, pyro- and ferroelectric properties [17,57]. Among the applications are transducers and sensors, human health monitoring systems, biomedical materials, polymeric electrolytes, and filtration membranes [91,130,131].

Portable and wearable PE nanogenerators (PENG), which can convert mechanical strain to electrical energy, are the attractive field of research in the literature. Wearable and implantable flexible PENG for physiological signal sensing and studying sleep quality are among the most anticipated areas of examinations. PVDF cable sensors or PVDF-sensor-array-integrated mattresses have been developed and can record cardiac and respiratory actions while the subject is in a lying posture [90,149].

Other distinctive features include its small size, light weight, ease of use, low cost, portability, and the ability to harvest energy efficiently from heat [17], light [18], mechanical vibration [150], wind energy [67], sonic waves [149], fluidics [90], and bio-motions [135] present in the environment. The practicability of the PNGs is already verified by powering different devices such as light-emitting diodes (LEDs) [151], liquid crystal displays (LCDs) [152], speakers, watches [153], medical imaging [117], telecommunication, and ultrasonic devices [114].

For instance, it would be fascinating having muscles spread in a hosting material, such as in a bat’s wing, to sense and actuate in the latter case. Little work has been conducted in the perspective mentioned above; electrospun muscles that find their actuation capability in environmental pH changes were successfully made of polyacrylonitrile [135].

As reviewed in [154], PLLA-based composites have been researched extensively in order to be applied in the field of orthopedic regenerative engineering [155,156]. Although PLA PE properties have been proven [64], the focus of the researchers has been on improving the suitability of the patches for the desired application, accompanied by no dynamic environment and without mentioning the contribution of PE properties of fillers or PLA itself.

### 5.1. The Future Portrait of Tissue Regeneration

As opposed to ferroelectric ceramics, ferroelectric polymers are profoundly flexible, biocompatible, and processable, making them exciting candidates for wearable and implantable electronics for human bodies. Relating to this, PVDF and its copolymer, because of their biocompatibility, high chemical resistance, light weight, flexibility, high PE properties, and economic considerations, are the surrogate choices to fabricate nanogenerators. The next revolution in PE properties emerged when decent PE materials such as Organic PE materials and synthetic polymers called “piezo polymers” were found [30,157,158,159,160].

In contrast to common PE materials such as lead zirconia titanate (PZT) [161] and polyvinylidene flurried (PVDF) [162], PLA is less studied, and thus there is a long way to go to utilize its properties. Despite most ceramic PE devices requiring an external foreign electrical or magnetic field, after fabrication, PLA asks for no electrical poling treatment to show the piezo-response from itself [117,153].

In materials with PE performance, displacement is generated without an external electric field; regardless, there are barriers to control the stimulus [163]. The importance of the interface between the bioelectronic devices and human bodies highlights the biosafety concerns. The biodegradability of organic and synthetic materials is one of the greatly desired features, which should be considered to prevent the additional surgical procedure. With this in mind, biocompatible and biodegradable polymers, which are also environmentally friendly, are impressive materials [17,24,85,164].

The process, which leads the biological PEs mainly mimicking, consists of stretching the intrinsic piezo materials that can degrade in similar body conditions. The benefit of PE in tissue regeneration might be because of the fact that polarized polymers show more significant protein adsorption and enhanced cellular adhesion and proliferation [165,166].

Currently, the high demand for stretchable and wearable electronics has attracted lots of attention itself. Innovative stretchable PE materials convert the forms of energy to each other by bending repetitions, folding, or stretching without loss of performance and are highly beneficial in wearable and portable electronics such as touch screens, wearable sensors, military garment devices, and biomedical applications [167].

As a matter of composition, TPS, a promising biodegradable material with abundance, high ductility, and low cost, has drawn attention; hence, it is used to blend with PLA [168]. However, these two hydrophobic materials are incompatible, leading to phase separation [169]. Because TPS contains hydroxyl group-rich plasticizer and starch, it is highly hygroscopic and sensitive to moisture absorption when stored at a high temperature; this is a barrier to mat fabrication and causes poor mechanical properties and sticky surface [122].

### 5.2. Tissue Engineering and Regenerative Medicine

The main pillars of TE are cells, scaffolds, and biologically active signaling molecules. Artificial patches act to mimic the ECM and their biocompatibility with the appropriate fiber diameter and pore size to make cell attachment and migration possible. Sufficient surface area and surface chemistry to facilitate cell adhesion, growth, migration, and differentiation, robust mechanical properties, and a biodegradation rate similar to the regeneration rate of the tissue being engineered are the factors that should be considered [170].

Demanded power-based electric generators are top-rated in bio-applications because of they are lightweight to wear in addition to their in vivo performance despite lab-on-chip devices. Nanomaterial PEs can be integrated with composite polymer and fibers. However, a durable, flexible, and wearable power generator that can generate an adequate electric field from human motions must be produced [167].

Scaffolds manufactured from polymer-nanoparticle composites can be performed with two distinct strategies; first, incorporating the nanoparticles into the polymer matrix, and second, including instinct polymers [171]. Utilizing metal nanoparticles is more common because of their unique electrical, optical, and catalytic properties [172]. For instance, combining gold and silver nanoparticles and carbon nanotubes to various polymers in the ES process has been widely researched [171,172,173].

#### 5.2.1. PLA Composite

**Figure 11:**
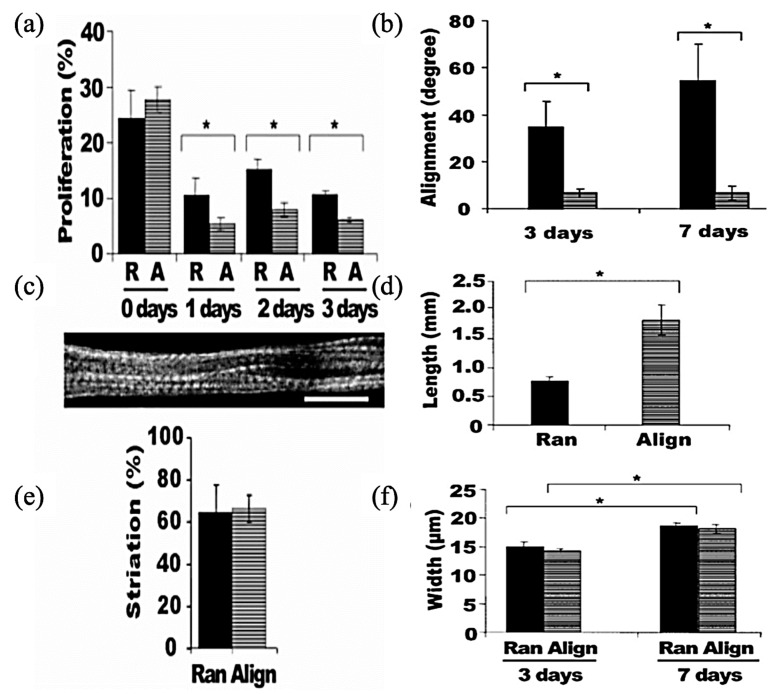
Quantification of myoblast proliferation and myotube striation on aligned nanofibrous scaffolds: (**a**) BrdU incorporation for cell proliferation (R, ran; A, align); (**c**) Immunofluorescence staining of anti-MHC showing a striated myotube on an aligned nanofibrous scaffold (scale bar, 20 μm); (**e**) Quantification of the percentage of striated cells after 7 days. Asterisks indicate a statistically significant difference (* *p* < 0.05); (**b**) The angle of myotube alignment in reference to nanofiber direction; (**d**) Myotube length after 7 days; (**f**) Myotube width after 7 days. Asterisks indicate a statistically significant difference (* *p* < 0.05); Reproduced from [174], with permission from Nano Letter; American Chemical Society, 2006.

As is evident in Figure 11, cultured murine C2C12 myoblast on electrospun PLLA fibers produced highly organized myotubes grown along the nanofibers [174]. ES can provide a way to fabricate dense and uniformly aligned packed skeletal muscle tissue, which greatly resembles the native skeletal muscle [175].

Freeman et al. [176] came up with PLLA gold-nanoparticle composites, and skeletal muscle TE was also investigated. This research concentrated on employing different proportions of gold nanoparticles in the PLLA matrix to study mechanical properties, electrical conductivity, and cellular behavior while lacking the ability to improve the fiber alignment and decrease the agglomeration of the conductive particles and solve the fiber fusion matter. The results show that although the cellular proliferation was low on their scaffolds, they showed that the Au Nps displayed no toxic effects; on the other hand, the mechanical properties of the stands were higher than skeletal muscles, which suggest that the lower Au NPs have to be used to match the mechanical properties of the tissue.

Schlatter et al. fabricated an electrospun PLA: Poly(glycerol sebacate) (PGS) composite and used a post-annealing process, showing an increase in fiber thickness and crystallinity percentage and an improvement in the mats’ mechanical properties. On the other hand, they have demonstrated that neither the composition nor the annealing process improves the hydrophilicity of PLA considerably. Matrigel coating of the samples causes adhesion of cardiomyocytes to both PLA pure and composite samples. At the same time, it is demonstrated that this coating would act as a possible point to reform the shape of the cells from a spherulitic physique to a cylindrical shape. Finally, they established the vascularization formation in PLA versus PLA: PGS composite as a matter of diffusion [177].

**Figure 12:**
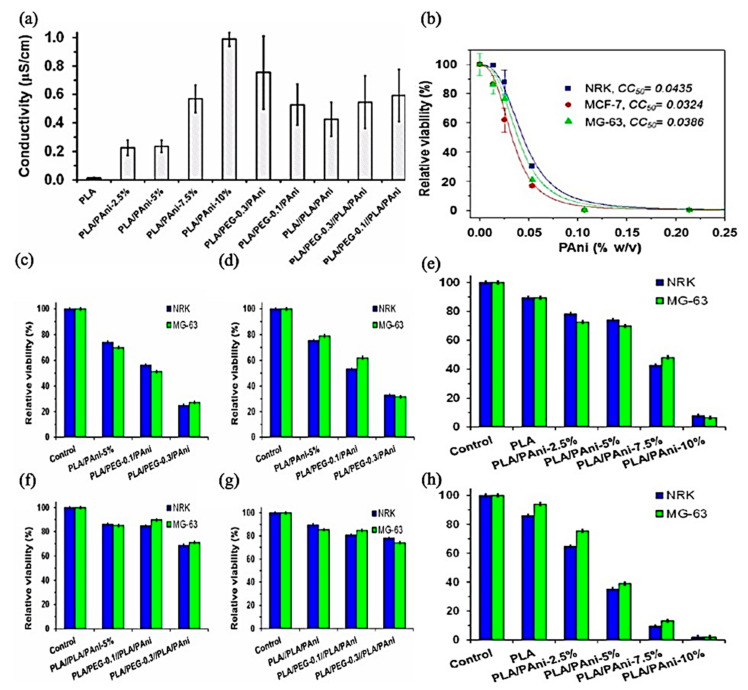
(**a**) Electrical conductivity of PLA, PLA/PAni, PLA/PEG/PAni, PLA//PLA/PAni, and PLA/PEG//PLA/PAni fibrous mats. (**b**) Cytotoxicity curve of PAni/DBSA for NRK, MCF-7, and MG-63 cells. (**e**,**h**) Biocompatibility of PLA/PAni fibers expressed as relative viability of NRK and MG-63 culture cells onto the fibrous mats after (**e**) 24 h (cell adhesion) and (**h**) 96 h (cell proliferation). Biocompatibility of (**c**,**d**) PLA/PEG/PAni and (**f**,**g**) PLA/PEG//PLA/PAni fibers expressed as relative viability of NRK and MG-63 culture cells onto the fibrous mats after (**c**,**f**) 24 h (cell adhesion) and (**d**,**g**) 96 h (cell proliferation); Adapted from [178], with permission from ACS Omega, 2019, https://pubs.acs.org/doi/abs/10.1021/acsomega.8b03411 (accessed on 25 August 2021), further permissions related to the material excerpted should be directed to the ACS.

Increasing the electroconductivity of polymeric materials is one of the requirements in the course of scaffold considerations. PANI is one of the promising materials to overcome this drawback. Although the PANI concentration increases electrical conductivity, its dose affects cytotoxicity, cell adhesion, and proliferation, as shown in Figure 12. PEG’s hydrophilicity decreases cell adhesion and proliferation [178].

**Figure 13:**
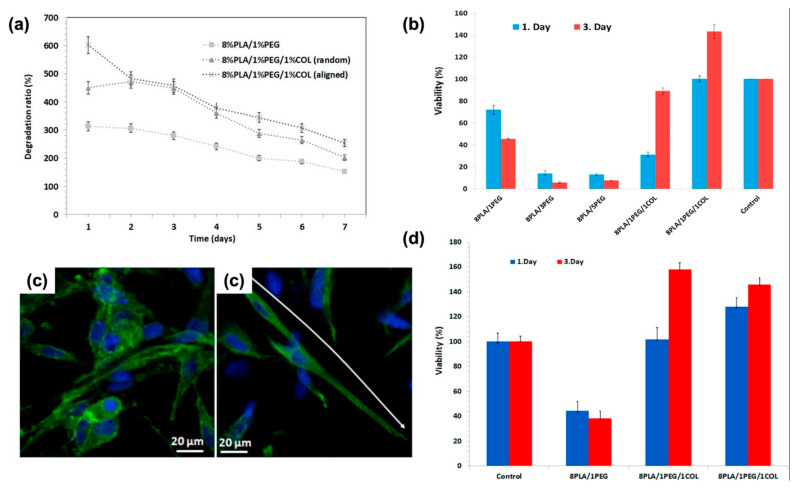
(**a**) The degradation behaviors of the patches after 7 days of incubation. (**b**) Cell viability graph of 2D H9C2 cells and nanofiber patches before and (**d**) after degradation. (**c**) Confocal images of random and (**left**) aligned (**right**) 8%PLA/1%PEG/1%COL nanofiber patches; Adapted from [179], with permission from Polymer testing; Elsevier, 2020.

Studying a PLA: PEG: COLLAGEN composite revealed that increasing the PEG concentration causes a promotion in fiber diameter while it would increase cell death. The research also studied the impact of aligned mats on mechanical and biological issues; on the one hand, the alignment would increase the tensile strength and decrease the swelling ratio at the same time and, on the other hand, cause homogeneous cardiomyocyte cell growth, as it is visible in Figure 13. On the contrary, the addition of collagen improves both cell proliferation and viability [179].

**Figure 14:**
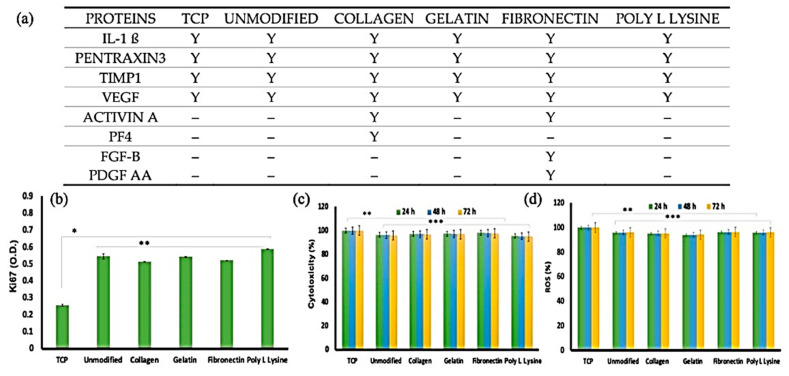
(**a**) Expressed proteins with a major role in cardiac fibroblast growth and differentiation. (**b**) Ki67, a cellular proliferation marker, was recorded at higher expression levels in cells cultured on the unmodified and protein-modified porous PLLA scaffolds than in the tissue culture plastic (TCP) substrates. The expression difference was a significant 2-fold increase in the fiber scaffolds than TCP. These observations support the enhanced compatibility the scaffolds share with the cells than conventional culture ware (*n* = 3, * *p* < 0.05, ** *p* < 0.01). (**c**) Cytocompatibility and reactive oxygen species (ROS) generation analysis of AHCF cultured on unmodified and protein-functionalized PLLA scaffolds. (**c**) No significant toxicity was observed with any of the scaffold variants. AHCF cultured on TCP were used as positive controls. (**d**) The AHCF cells cultured on the scaffolds did not show any noticeable ROS production, providing evidence that the scaffolds do not exert any stress on cells (*n* = 3, ** *p* < 0.01, *** *p* < 0.001); Adapted from [180], with permission from Polymers; MDPI, 2020.

Modifying microfiber PLA scaffolds with various proteins was taken into consideration [180]; they coated collagen, gelatin, fibronectin, and Poly L lysine to the artificial mats, and their degradation rate up to 30 days was studied. An adult human cardiomyocyte adhesion and proliferation study revealed that the unmodified sample shows better cell adhesion, probably due to its rough topography. On the other hand, cytotoxicity results show us that the scaffolds do not exert any stress on the cells. The cell proliferation marker (Ki67) showed an improvement in proliferation kinetics, as shown in Figure 14.

#### 5.2.2. PLGA Composites

PLGA is a biopolymer with no piezo properties but a low degradation time. Zheng et al. fabricated a fibrous composite consistent with PDLA, PLGA, and CoFe_2_O_4_ nanoparticles (30 nm). Beading is apparent in fabricated samples with lower concentrations related to viscosity. Thicker fabricated fibers in higher concentrations result in a more uniform morphology. The presence of magnetoelectric CoFe_2_O_4_ particles motivates the local electric field. The existence of amorphous PLGA causes no XRD peak; in addition, because of semi-crystalline PDLA components, peak appearance is possible in DSC. In this research, the piezo-response is just ascribed to the PDLA feature [181].

Khan et al. seeded the human-induced pluripotent stem cells cardiomyocytes into the electrospun polylactide-co-glycolide (PLGA). They fabricated 50μm-thick aligned nanofibrous scaffolds and compared the outcome against conventional tissue culture plastic surfaces. The outcomes from this research revealed that the scaffolds have the potential to align the CMs and provoke functionality changes of Calcium transients and gene expression through the appropriate stiffness of the mats [182].

**Figure 15:**
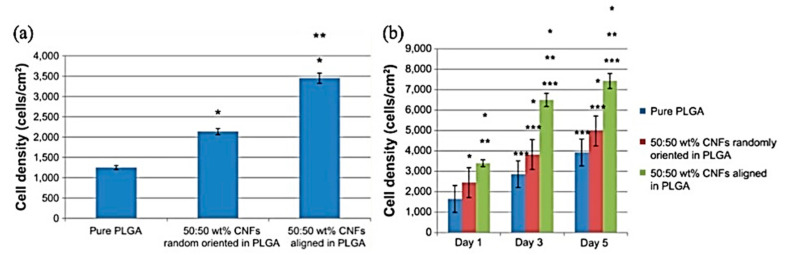
(**a**,**b**) Increased cardiomyocyte adhesion on aligned CNFs compared with randomly oriented CNFs in PLGA composites, (**a**) The adhesion time was 4 h. Seeding density 3500 cells/cm2. Data shown as the mean ± standard error of the mean (*n* = 3). * *p* < 0.01 compared with pure PLGA and ** *p* < 0.01 compared with 50:50 wt% CNFs randomly oriented in PLGA, (**b**) Seeding density 3500 cells/cm2. Data shown as the mean ± standard error of the mean (*n* = 3). * *p* < 0.01 compared with pure PLGA at respective time period; ** *p* < 0.01 compared with 50:50 wt% randomly oriented CNFs in PLGA at respective time period; *** *p* < 0.01 compared with previous time period, same sample; Adapted from [183], with permission from International Journal of Nanomedicine; Dove Medical Press, 2014.

Taking the PLGA and Carbon Nanofiber (CNF) composites to account, increasing the CNF content caused an enhancement in the conductivity and cytocompatibility of PLGA, in addition to cardiomyocyte adhesion and proliferation improvement. As shown in Figure 15, this study revealed an increase in the density of cardiomyocytes with an increase in CNF up to 25:75 wt.% PLGA: CNFs [183].

Asiri et al. fabricaterd an aligned 20 μm wide patter of 100 nm CNFs on the surface of PLGA with a 50:50 PGA: PLA weight ratio.They demonstrated that, on the one hand, the alignment of CNF increases the density of cardiomyocytes, and on the other hand, it promotes the longitudinal conductivity to 0.1 S/m and decreases the horizontal conductivity to 0.0025 S/m compared to randomly oriented fibers; these conductivities are similar to natural heart tissue [184].

In research from Gelmi and colleagues, they chlorine-doped PPy deposited on electrospun PLGA fibers and made a 3D and electrically conductive scaffold. They confirmed the biocompatibility of these scaffolds using cardiac progenitor cells and iPSCs. Later, using PPy, they coated PLGA to make a biocompatible EA patch for cardiogenic differentiation under electromechanical stimulation [185].

Furthermore, 3D PLGA-PANI aligned fibers were investigated by Hsiao et al. for the synchronous beating of cardiomyocytes. The fabricated scaffold increased the gap junction protein expression (Cx43) and troponin T. Isolated cell clusters with synchronous beatings formed from cardiomyocytes. The HCL-doped PANI promoted electrical conductivity, aroused positively charged cell membrane proteins, and advanced cell adhesion [186].

To yield electrically conductive hydrogels, attempts at incorporating PANI with different hydrogel and polymers have been triggered recently. Cui et al. fabricated a hydrogel composed of polylactide-poly(ethylene glycol)-polylactide (PLA-PEG-PLA) copolymer coated with tetra aniline (with carboxylate modification) to culture cardiomyocytes, fibroblasts, and osteoblasts. Applied electrical stimulation to the cultured cells on samples enhanced cell proliferation [187,188].

## 6. Conclusions

ES was marked as the best procedure to induce PE in PLA ECM mimicking mats among the numerous fabrication procedures. This was attributed to the high shear force applied to the chains that stimulate C=O dipoles to be oriented even in an amorphous structure. Although PLA was not a ferroelectric polymer, it showed PE, even in an amorphous state while inducing even a partially long-range ordering of the chains through thermo and/or mechanical work, which ought to increase the PE outcome. Regarding the fabrication of interconnected porous mats, in addition to what has already been denoted in the literature, it was shown in the above-discussed script that a micro-aligned mat, or in a better mode, nanoscale fibers had a tremendous potential to be used as artificial organs or at least a base for migration of the immature cells to the cells which were dead or could not perform their duty. The origin of the PE was described based on a non-centrosymmetric structure, and PLA had this non-centrosymmetry structure even in its amorphous state due to its chiral conformation. Tolerating the piezoelectric outcome required a semicrystalline state, which was induced by various PLA crystalline phases of α, α’, β, and γ, all showing the PE within the lack of centrosymmetric. Another perspective that should be taken to account was the preferential crystalline planes; any texture induced by mechanical process improved the piezoelectric performance, as evidenced by 2D XRD narrations. Coming to the point, PLA biomaterial is going to be used in numerous aspects of TE, and numerous efforts have been made to improve PLA’s hydrophilicity; however, it has been observed that it is not that essential for cell growth compared to tolerating its mechanical properties, such as its brittleness and stiffness, to be used in TE applications. Since the activation and use of the P requires a type of applied pressure or mechanical work to scaffolds, the mat deformability is a matter of future attention that should be tuned simultaneously with the PE outcome.

## Figures and Tables

**Table 1:** PLA crystalline phase information.

Crystalline Phase	Unit Cell	Conformation	Formation Condition	References
α	Orthorhombic a = 9.95 Å, b = 6.25 Å, c = 8.8 Å	10_3_ left-handed helix	Crystalizes above 120 °C	[104]
α’	Pseudo-hexagonal a = b= 6.2 Å, c = 28.8 Å	10_3_ distorted helix	Crystalizes below 120 °C	[105]
β	Trigonal a = b = 10.52 Å, c = 8.8 Å	3_1_ left-handed helix with frustrated structure	Drawing ratio ≥ 6 T ≈ T_melting_	[106]
γ	Orthorhombic a = 9.95 Å, b = 6.25 Å, c = 8.8 Å	3_1_ antiparallel left-handed helix	Epitaxial crystallization on hexamethylbenzene (HBM) substrate	[107]

**Table 2:** FTIR C=O absorption wavenumbers corresponding to their conformation state, produced from the data available in [120], Marcromulecules, ACS, 2009.

Conformation	g-g	t-g	g-t	t-t
**Wavenumber (cm^−1^)**	1777	1767	1758	1749
**δ (Phase)**	0°	72°	108°	180°
**Helix type**	-	5_1_	10_3_	2_1_

**Table 3:** A comparison of different piezoelectric constant of materials; Reproduced from [127], with permission from Advanced Materials; Wiley-VCH, 2018.

Piezoelectric Material	Material Type	Piezoelectric Constant
**PZT-5H**	Anisotropic (Orthorhombic), Ceramic	d_33_ = 593 (pC/N), d_31_ = −274 (pC/N)
**AIN**	Anisotropic, Ceramic	d_33_ = 3–6 (pC/N), d_31_ = −2 (pC/N)
**Quartz**	Anisotropic (Orthorhomic), Single crystal	d_11_ = 2.3 (pC/N), d_14_ = −0.67 (pC/N)
**ZnO**	Anisotropic, Crystal	d_33_ = 6–13 (pC/N), d_31_ = −5 (pC/N)
**BaTiO_3_**	Anisotropic (Orthorhombic), Ceramic	d_33_ = 190 (pC/N), d_31_ = −78 (pC/N),
**LiNbO_3_**	Anisotropic (Orthorhombic), Ceramic	d_33_ = 16 (pC/N), d_31_ = −1 (pC/N)
**PMN-PT**	Anisotropic, Single Crystal	d_33_ = 2000-3000 (pC/N)
**GaN**	Anisotropic, Crystal	d_33_ = 2–4 (pC/N), d_31_ = −1.5 (pC/N)
**PVDF**	Anisotropic, Polymer	d_33_ = −33 (pC/N), d_31_ = 23 (pC/N),
**PLLA**	Anisotropic (Transversely Isotropic), Polymer	d_14_ = 6–12 (pC/N), d_33_ = 3.08 (pC/N)
**β -Glycine**	Anisotropic, β-Crystal	d_16_ = 196 (pm/V)
**Collagen**	Anisotropic, Non-oriented	d_14_ = 0.1 (pm/V)
**Silk**	Anisotropic, Semi-Crystalline, Drawing Ratio = 2.7	d_16_ = −1.5 (pC/N)
**Peptide Nanotubes**	Self-assembly process of diphenylalanine	d_15_ = 60 (pm/V)
**Graphene**	Single-layer	d_33_ = 1.4 (nm/N)

## Data Availability

The raw/processed data required to reproduce these findings cannot be shared at this time as the data also form part of an ongoing study.
